# Comparative analysis of SCGN expression patterns in the anterior pituitary gland of mouse, pig, and human by single‐cell transcriptomics

**DOI:** 10.1111/jne.70249

**Published:** 2026-08-23

**Authors:** Cheng‐Cheng Wang, Qi‐Lin Zhang, Zhen‐Le Zang, Hua‐Chun Yin, Yu Pan, Yi‐Fei Yu, Yi‐Ming Li, Zhao‐Yun Zhang, Song Li, Hui Yang

**Affiliations:** ^1^ Chongqing Institute for Brain and Intelligence, Guangyang Bay Laboratory Chongqing China; ^2^ Department of Neurosurgery and National Center for Neurological Disorders Huashan Hospital, Shanghai Medical College, Fudan University Shanghai China; ^3^ Department of Neurosurgery Xinqiao Hospital, Army Medical University Chongqing China; ^4^ Chongqing Medical University Chongqing China; ^5^ Department of Endocrinology and Metabolism Huashan Hospital, Fudan University Shanghai China

**Keywords:** anterior pituitary gland, expression, secretagogin, single‐cell transcriptomics, species specificity

## Abstract

Secretagogin (SCGN) is a calcium‐binding protein implicated in neuroendocrine secretion. However, its expression profile in the anterior pituitary gland and potential species‐specific differences remain poorly characterized. We analyzed scRNA‐seq data from the pituitary glands of mice, rats, pigs, and humans, then combined it with validation by immunostaining to systematically elucidate the expression and distribution patterns of SCGN in the anterior pituitary gland. SCGN exhibited distinct species‐specific expression patterns. In mice, SCGN transcripts were predominantly enriched in PIT1‐lineage lactotropes and SF1‐lineage gonadotropes, with immunofluorescence confirming high colocalization with LH, FSH, and PRL. In pigs, SCGN was primarily expressed in TPIT‐lineage corticotropes, and immunofluorescence colocalization analysis confirmed the highest colocalization rate with ACTH. In humans, SCGN expression was confirmed in the anterior pituitary gland by immunohistochemistry, while scRNA‐seq revealed predominant enrichment of its transcripts in PIT1‐lineage lactotropes and somatotropes. Notably, comparative interspecies analysis indicated a high degree of similarity in SCGN enrichment within PIT1‐lineage cells (particularly lactotropes) between mice and humans. In contrast, SCGN expression was undetectable in the rat anterior pituitary gland at both transcriptional and protein levels. Together, our findings suggest that SCGN may not be an essential or universal regulator of anterior pituitary hormone secretion. Instead, this study provides an informative negative example that highlights the interspecies divergence of the pituitary secretory machinery among mammals.

## 1 INTRODUCTION

The pituitary gland is one of the most critical endocrine glands in mammals, and it can be divided into the anterior lobe and posterior lobe. The anterior pituitary gland comprises the pars distalis, pars tuberalis, and pars intermedia. The endocrine functions of the anterior pituitary gland are precisely regulated by hypothalamic neurons, which secrete releasing hormones into the hypothalamic‐hypophyseal portal system. These hormones modulate five major cell populations in the anterior pituitary: corticotropes (ACTH), somatotropes (GH), lactotropes (PRL), thyrotropes (TSH), and gonadotropes (LH and FSH). During development, the differentiation of these cell types is governed by specific transcription factor cascades: PIT1 directs the differentiation of somatotropes, lactotropes, and thyrotropes; TPIT determines corticotrope differentiation; and SF‐1 regulates gonadotrope differentiation.^1^, ^2^, ^3^ Through the secretion of various hormones, anterior pituitary cells orchestrate essential physiological processes such as growth, development, behavior, and reproduction. They also influence internal homeostasis and regulate other endocrine organs. The dynamic balance of their secretory activity is fundamental to maintaining endocrine homeostasis and is closely associated with multiple major endocrine disorders.^4^

In recent years, secretagogin (SCGN), a calcium‐binding protein, has garnered significant attention for its role in neuroendocrine regulation. As a member of the EF‐hand superfamily, SCGN is a highly conserved protein containing six EF‐hand domains. It is highly expressed in pancreatic β‐cells but is also found in the central nervous system and gastrointestinal enteroendocrine cells.^5^, ^6^, ^7^ Recent studies indicate that SCGN regulates secretory granules dynamics by interacting with the core exocytotic SNARE protein SNAP‐25 and β‐actin, thereby serving as a key regulator in the vesicular secretion of hormones such as insulin, corticotropin‐releasing hormone, and oxytocin.^8^, ^9^, ^10^, ^11^, ^12^

Accumulating evidence shows that SCGN is expressed in various neuronal and non‐neuronal cell types with exocytotic activity, including pituitary adenohypophyseal cells, hypothalamic cells, pancreatic, intestinal and thyroid cells.^13^, ^14^, ^15^ However, its expression and function within the anterior pituitary gland remain largely unexplored. Therefore, this study aims to identify the cell types expressing SCGN in the anterior pituitary gland across different species (including mice, rats, pigs and humans) by employing scRNA‐seq analysis and immunostaining, and to provide a comparative expression atlas of SCGN that may help to assess whether its expression is evolutionarily conserved in the anterior pituitary and to what extent its function might be species‐restricted.

## 2 MATERIALS AND METHODS

### 2.1 Animals

In this experiment, 2‐month‐old C57BL/6 mice, Sprague–Dawley (SD) rats, and 3‐month‐old Bama pigs were housed under standard and appropriate conditions, maintained on a 12‐h light/12‐h dark cycle, with ad libitum access to food and water. Animals were anesthetized with an intraperitoneal injection of 1% sodium pentobarbital (50 mg/kg) and euthanized. All experimental procedures were conducted in accordance with the principles of laboratory animal care and were approved by the Ethics Committee (AMUWEC20245310).

### 2.2 Pituitary gland dissociation

Porcine pituitary glands were dissected immediately after euthanasia, and the pituitary glands were promptly dissected and placed in sterile culture dishes. Tissues were rinsed 1–2 times with ice‐cold DPBS (Thermo Fisher, 14,190,144), minced into 1–2 mm^3^ fragments, and digested enzymatically at 37°C in a shaking water bath. Cell suspensions were filtered using a 40 μm nylon cell strainer, and red blood cells were lysed using Red Blood Cell Lysis Solution (Thermo Fisher, 00–4333–57). Cells were washed, resuspended in DPBS containing 2% FBS, and kept on ice. Cell concentration and viability were assessed using an automated cell counter (Countstar) with Acridine Orange/Propidium Iodide (AO/PI) staining.

### 2.3 10× library preparation and sequencing

A single‐cell suspension was adjusted to a concentration of 700–1200 cells/μL. Single‐cell libraries were then generated using the 10× Genomics Chromium™ Controller system. Beads with unique molecular identifier (UMI) and cell barcodes were loaded close to saturation, so that each cell was paired with a bead in a Gel Beads‐in‐emulsion (GEM). After exposure to cell lysis buffer, polyadenylated RNA molecules hybridized to the beads. Beads were retrieved into a single tube for reverse transcription. On cDNA synthesis, each cDNA molecule was tagged on the 3′ end (that is, the 5′ end of a messenger RNA transcript) with UMI and cell label indicating its cell of origin. Briefly, 10× beads were then subjected to second‐strand cDNA synthesis, adaptor ligation, and universal amplification. Sequencing libraries were prepared using randomly interrupted whole‐transcriptome amplification products to enrich the 3′ end of the transcripts linked with the cell barcode and UMI. All the remaining procedures including the library construction were performed according to the standard manufacturer's protocol (Chromium Single Cell 3′ v3.1). Sequencing libraries were quantified using a High Sensitivity DNA Chip (Agilent) on a Bioanalyzer 2100 and the Qubit High Sensitivity DNA Assay (Thermo Fisher Scientific). The libraries were sequenced on Illumina Xplus/DNBSEQ‐T7 using 2 × 150 chemistry.

### 2.4 Bioinformatic analyses of scRNA‐seq data

In addition to the scRNA‐seq data generated from porcine pituitaries in this study, we obtained publicly available scRNA‐seq datasets for the pituitary glands of other species. These included data from C57BL/6 mice^16^ (GSM4594334, GSM4594335, GSM4594336, GSM4594338, GSM4594339) and SD rats^17^ (GSE132224) from the NCBI GEO (Gene Expression Omnibus) database, as well as human pituitary data^18^, ^19^ (HRA002436, HRA003483) from the GSA (Genome Sequence Archive).

The UMI count matrices were obtained, imported into R, and analyzed using the Seurat package (10.1016/j.cell.2021.04.048). Low‐quality cells were removed based on the following criteria. (i) Cells with relatively high mitochondrial percentage (mitochondrial reads >10%) were removed. (ii) The thresholds for acceptable numbers of detected genes and UMIs per cell were determined by outliers in the joint distribution of unique UMIs and detected genes across cells. For the mice scRNA‐seq data, cells were filtered by retaining those with RNA counts between 200 and 40,000, fewer than 6000 detected genes. The rat dataset was subset to include cells with 200–6000 detected genes and total RNA counts below 40,000. For human samples, the merged seven datasets were subset using: nCount_RNA < 75,000, 500 < nFeature_RNA < 8000. For pig samples, cells were retained if they expressed between 100 and 7500 genes.

The datasets were log‐normalized, and highly variable genes were calculated with the FindVariableFeatures function. Cell cycle effects were regressed out using the ScaleData function. PCA was calculated using the RunPCA function, after which the RunHarmony function was used. The Harmony reduction was then used in FindNeighbors (dimensions of mice and rat dataset = 1:20 and dimensions of human and pig dataset = 1:30), FindClusters (resolution of mice and rat dataset = 0.4, resolution of human data = 0.3 and resolution of pig data = 0.5). Cell markers were used to annotate the clusters from the literature for pituitary cells.^20^, ^21^, ^22^, ^23^

### 2.5 Real‐time quantitative PCR

Total RNA was extracted from tissue samples using the Trizol method. Subsequently, Takara's PrimeScript™ RT reagent kit with gDNA Eraser (Perfect Real Time) (RR047A) was used for reverse transcription, and the resulting cDNA was stored at −20°C for future use. The qPCR reaction was performed using the SYBR Green Premix Pro Taq HS qPCR Kit (AG11701) from Accurate Biotechnology on the Agilent AriaMX Real Time PCR System. Reaction procedure: Pre‐denaturation at 95°C for 30 s; then 40 cycles of 95°C denaturation for 5 s and 60°C annealing extension for 30 s; perform melting curve analysis after the cycle ends to verify the specificity of the amplified product. Using GAPDH as an internal reference, the relative expression level of SCGN was calculated using the 2^(−∆∆Cq)^ method. All experiments were independently repeated at least three times. SCGN primers used in the experiment: SCGN‐F: TCTTTCGCCTGGAAACTCCC and SCGN‐R: AAGAGCCAGAATCCTTGCCA.

### 2.6 Immunofluorescence

Immunofluorescence staining was performed using an immunofluorescence staining kit (AFIHC023) from AiFang Biological. Frozen sections (20 μm) of mouse and pig pituitary glands, as well as rat pancreas, hypothalamus, and pituitary gland were prepared. Frozen sections were thawed to room temperature, rinsed with PBS, and permeabilized with 0.3% Triton X‐100. Antigen retrieval was performed using Tris‐EDTA buffer. After blocking endogenous peroxidase and non‐specific binding sites with peroxidase inhibitors and 5% BSA, sections were incubated overnight at 4°C with the mouse monoclonal SCGN (F‐9) antibody (Santa Cruz, sc‐374355, RRID: AB_10989370). As negative controls, adjacent sections were processed in parallel by omitting the primary antibody (replaced with PBS containing 5% BSA). Polymer‐HRP conjugated goat anti‐mouse/rabbit secondary antibody was applied and incubated at room temperature for 1 h. Signal was detected using tyramide signal amplification (TSA) with TYR‐570 dye. The sections then underwent a second round of antigen retrieval, peroxidase blocking, and incubation with 5% BSA. They were subsequently incubated overnight at 4°C with one of the following primary antibodies against pituitary hormones: mouse monoclonal LH antibody (Santa Cruz, sc‐374017, RRID: AB_10917560) and rabbit monoclonal LH antibody (abcam, ab150416, RRID: AB_2928120), rabbit monoclonal FSH antibody (abcam, ab281562, RRID: AB_2928119), rabbit monoclonal ACTH antibody (abcam, ab74976, RRID: AB_1280736), rabbit polyclonal GH antibody (Proteintech, 55243‐1‐AP, RRID: AB_11182710), rabbit monoclonal PRL antibody (abcam, ab183967, RRID: AB_2814839), or rabbit monoclonal TSH antibody (absin, abs145553, RRID: AB_3720121). A second round of staining was performed using Polymer HRP conjugated goat anti‐mouse/rabbit secondary antibody and TYR‐520 dye. Nuclei were counterstained with DAPI (Biosharp, BL105A); sections were mounted using UltraCruz® Aqueous Mounting Medium with DAPI (Santa Cruz, sc‐24941). Images were captured using an Olympus BX63 fluorescence microscope to quantify the colocalization rate of SCGN with hormonal signals.

### 2.7 Immunohistochemistry

Three human adult anterior pituitary gland tissues were obtained from cadaveric organ donors without evidence of any endocrine disease as in our previous study.^23^ Following deparaffinization and rehydration, heat‐induced epitope retrieval (HIER) was performed by submerging the slides in antigen unmasking solution (Solarbio). After blocking endogenous peroxidase and nonspecific binding sites (0.3% H_2_O_2_ and 5% normal goat serum, sequentially), primary antibodies (anti‐SCGN, Santa Cruz, sc‐374355) were applied at 4°C overnight. Slides were incubated with Dako REAL EnVision HRP rabbit/mouse (belong to K5007, DAKO, Glostrup, Denmark) at RT for 20 min, followed by treatment with Dako REAL DAB + CHROMOGEN and Dako REAL substrate buffer (belong to K5007, DAKO, Glostrup, Denmark) to visualize staining signals under light microscopy, and finally counterstained using hematoxylin solution. Stained slides were scanned using Ocus (Grundium, Tampere, Finland).

### 2.8 Statistical analyses

For each experimental animal, five biological replicates were used. For each pituitary sample, three tissue sections were analyzed and 3–5 fields of view per section were selected based on tissue size. The co‐localization ratio (number of SCGN‐positive cells colocalizing with a given hormone / total number of SCGN‐positive cells) was calculated for each field individually. The ratios from all fields of the three sections were then averaged to obtain a single value per animal, and group data are presented as mean ± SEM (*n* = 5).

All colocalization percentages were arcsine square‐root transformed using the formula Y = arcsin(sqrt(Y/100)) in GraphPad Prism 9.0.0 (Transform function) before statistical analysis. One‐way ANOVA with Tukey's post‐hoc test was performed on arcsine square‐root transformed data. For datasets that remained heteroscedastic after transformation (male pig), Welch's ANOVA was additionally performed as a sensitivity analysis; the significance outcomes were unchanged. For clarity of interpretation, untransformed percentages are presented in all figures. The F statistic, degrees of freedom, and p values are reported in the figure legends. For rats (no SCGN expression detected) and humans (no quantitative IHC comparison across cell types), ANOVA was not applicable. Statistical significance was defined as *p* < .05.

## 3 RESULTS

### 3.1 SCGN is enriched in gonadotropes and lactotropes in the mouse anterior pituitary gland

**FIGURE 1:**
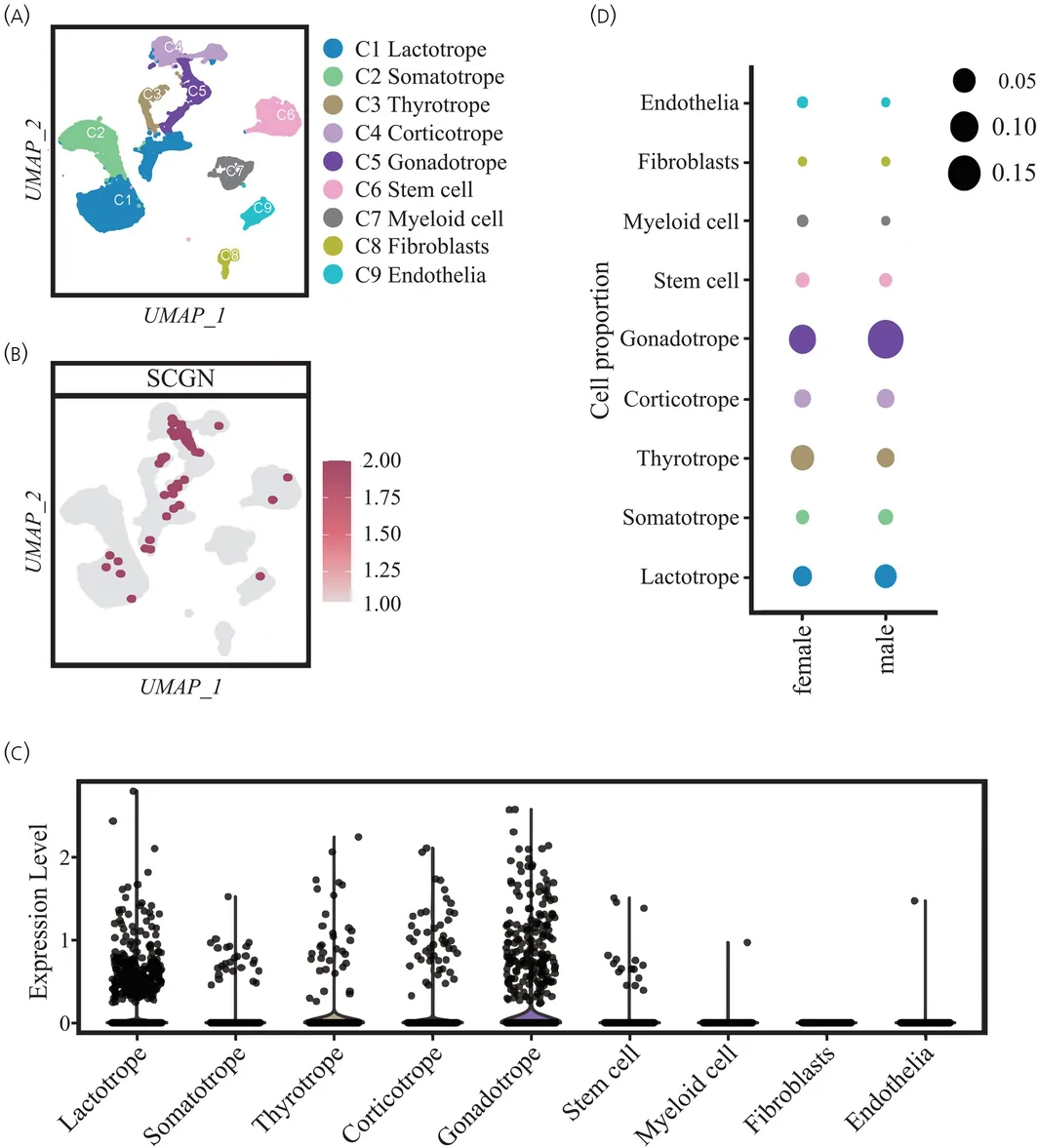
scRNA‐seq data analysis on the expression of SCGN in the anterior pituitary gland of mice (*n* = 5). A: Anterior pituitary gland cell clusters identified by genetic markers and visualized with UMAP. B: SCGN in the anterior pituitary gland of mice at the cell level. C: Expression of SCGN in 9 types of mice anterior pituitary gland cells. D: SCGN positive cells of male and female mice (2 female, 3 male). Circle represents the cell number percentage.

By integrating publicly available scRNA‐seq data of the pituitary gland from C57BL/6 strain mice, we annotated and clustered cell populations based on the expression signatures of key transcription factors, categorizing them into major types including lactotropes, gonadotropes, somatotropes, thyrotropes, and corticotropes (Figure 1A). Enrichment analysis of SCGN transcript expression revealed that SCGN is primarily enriched in lactotropes and gonadotropes, with minor distribution observed in somatotropes, thyrotropes, and corticotropes (Figure 1B,C). We further quantified the proportion of SCGN‐expressing cells within each anterior pituitary cell type in female and male mice. The proportions were similar between sexes, and the majority of SCGN‐positive cells were gonadotropes and lactotropes (Figure 1D).

**FIGURE 2:**
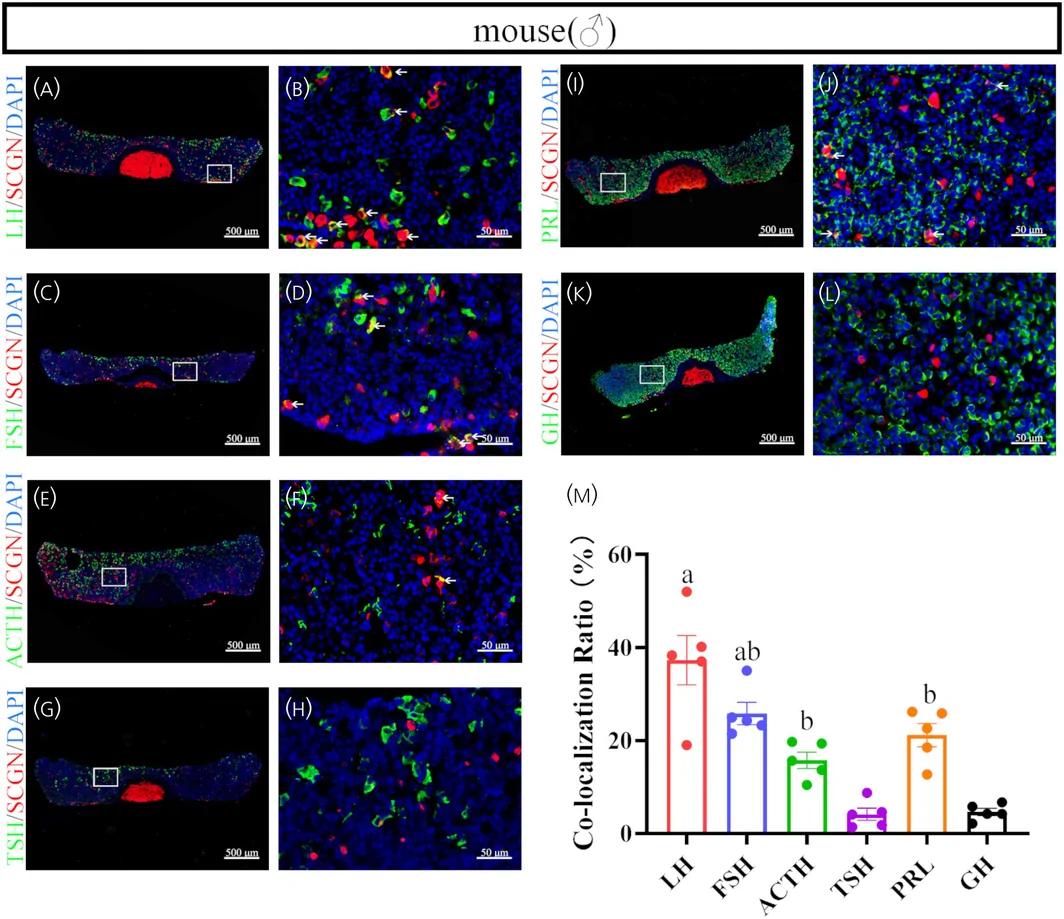
Immunofluorescence colocalization assays of SCGN in the anterior pituitary gland of 2‐month‐old male C57BL/6 mice (*n* = 5). A‐B: Colocalization of SCGN (red) and LH (green) in the anterior lobe (A), with a higher‐magnification view of the boxed area showing colocalization (arrows) (B). C‐D: Colocalization of SCGN (red) and FSH (green) in anterior lobe (C), with a higher‐magnification view of the boxed area showing colocalization (arrows) (D). E,F: Colocalization of SCGN (red) and ACTH (green) in anterior lobe (E), with a higher‐magnification view of the boxed area showing colocalization (arrows) (F). G‐H: Localization of SCGN (red) and TSH (green) in anterior lobe (G), with a higher‐magnification view of the boxed area showing the detailed localization (H). I‐J: Colocalization of SCGN (red) and PRL (green) in anterior lobe (I), with a higher‐magnification view of the boxed area showing colocalization (arrows) (J). K,L: Localization of SCGN (red) and GH (green) in anterior lobe (K), with a higher‐magnification view of the boxed area showing the detailed localization (L). Scale bars: 500 μm (A, C, E, G, I, K), 50 μm (B, D, F, H, J, L). Strong SCGN immunoreactivity observed in the posterior lobe.M: Quantitative analysis of colocalization rates between SCGN and individual anterior pituitary gland hormones in male mice. Data in the figure are mean ± SEM. Different letters above bars indicate significant differences (*p* < .05), bars sharing the same letter are not significantly different. One‐way ANOVA revealed a significant main effect of hormone type on colocalization rate (F (5, 24) = 27.75, *p* < .0001).

**FIGURE 3:**
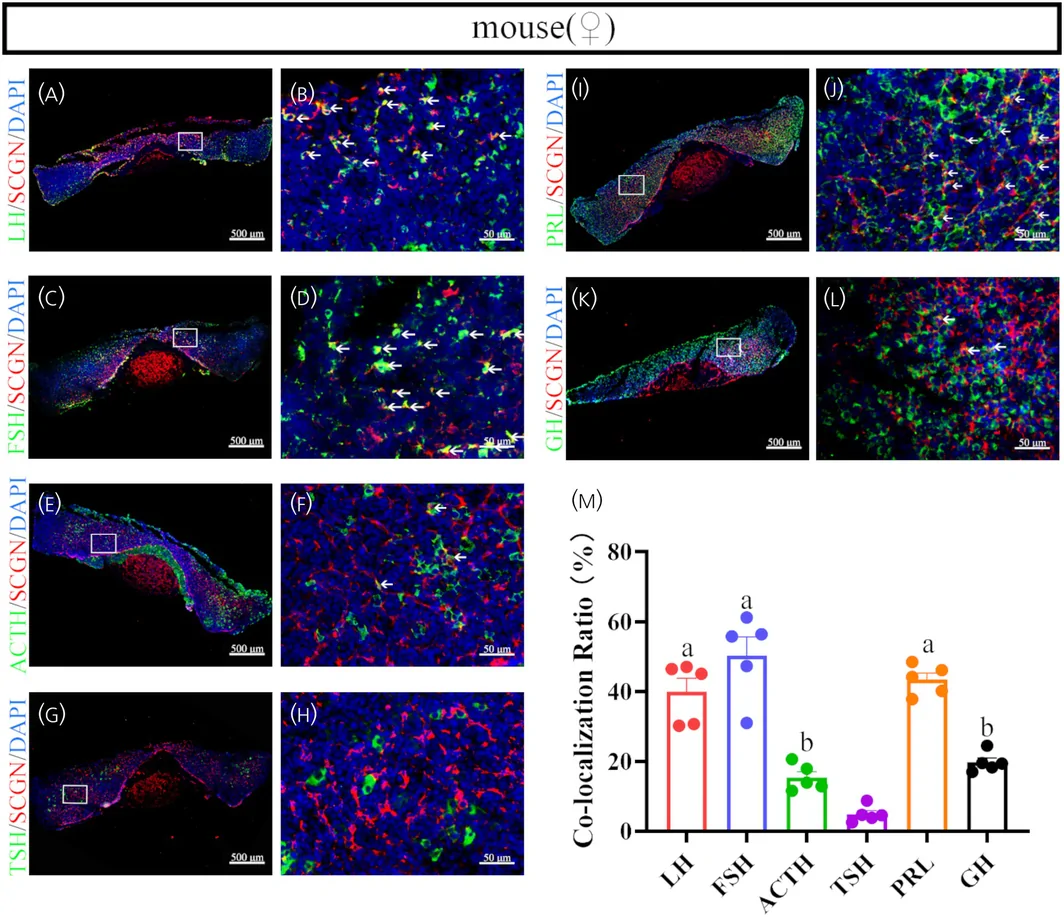
Immunofluorescence colocalization assays of SCGN in the anterior pituitary gland of 2‐month‐old female C57BL/6 mice (*n* = 5). A,B: Colocalization of SCGN (red) and LH (green) in the anterior lobe (A), with a higher‐magnification view of the boxed area showing colocalization (arrows) (B). C,D: Colocalization of SCGN (red) and FSH (green) in anterior lobe (C), with a higher‐magnification view of the boxed area showing colocalization (arrows) (D). E,F: Colocalization of SCGN (red) and ACTH (green) in anterior lobe (E), with a higher‐magnification view of the boxed area showing colocalization (arrows) (F). G,H: Localization of SCGN (red) and TSH (green) in anterior lobe (G), with a higher‐magnification view of the boxed area showing the detailed localization (H). I,J: Colocalization of SCGN (red) and PRL (green) in anterior lobe (I), with a higher‐magnification view of the boxed area showing colocalization (arrows) (J). K,L: Colocalization of SCGN (red) and GH (green) in anterior lobe (K), with a higher‐magnification view of the boxed area showing colocalization (arrows) (L). Scale bars: 500 μm (A, C, E, G, I, K), 50 μm (B, D, F, H, J, L). Strong SCGN immunoreactivity observed in the posterior lobe. M: Quantitative analysis of colocalization rates between SCGN and individual anterior pituitary gland hormones in female mice. Data in the figure are mean ± SEM. Different letters above bars indicate significant differences (*p* < .05), bars sharing the same letter are not significantly different. One‐way ANOVA revealed a significant main effect of hormone type on colocalization rate (F (5, 24) = 46.66, *p* < .0001).

Considering the potential role of SCGN in regulating the secretion of pituitary hormones, we performed double immunofluorescence staining to analyze the colocalization of SCGN with specific hormone markers (e.g., LH, FSH, GH, PRL, ACTH, TSH) in the anterior pituitary gland tissues from 2‐month‐old female and male C57BL/6 mice (Figure 2A–L and Figure 3A–L). In the anterior pituitary gland, SCGN is expressed in the cytoplasm of adenohypophyseal cells. The various subtypes of pituitary hormone‐secreting cells display distinct distribution patterns. Quantitative analysis of SCGN colocalization with various anterior pituitary hormones was conducted to evaluate its expression distribution. In males, SCGN showed the highest colocalization with LH (37.293% ± 11.830%), FSH (25.808% ± 5.322%), moderate rates were observed with PRL (21.176% ± 5.647%) and ACTH (15.762% ± 3.914%), while colocalization was minimal with GH (4.618% ± 1.747%) and TSH (4.135% ± 2.928%) (Figure 2M). In females, SCGN frequently colocalized with LH (39.922% ± 8.655%), FSH (50.346% ± 11.917%), and PRL (43.367% ± 4.336%). Lower colocalization rates were found with ACTH (15.381% ± 3.763%), GH (19.747% ± 2.859%), and TSH (4.897% ± 2.337%) (Figure 3M). The immunofluorescence colocalization results indicated that SCGN was mainly distributed in gonadotropes and lactotropes in the mouse anterior pituitary gland, which are highly consistent with the scRNA‐seq results.

### 3.2 SCGN is predominantly expressed in corticotropes in the male porcine anterior pituitary gland

**FIGURE 4:**
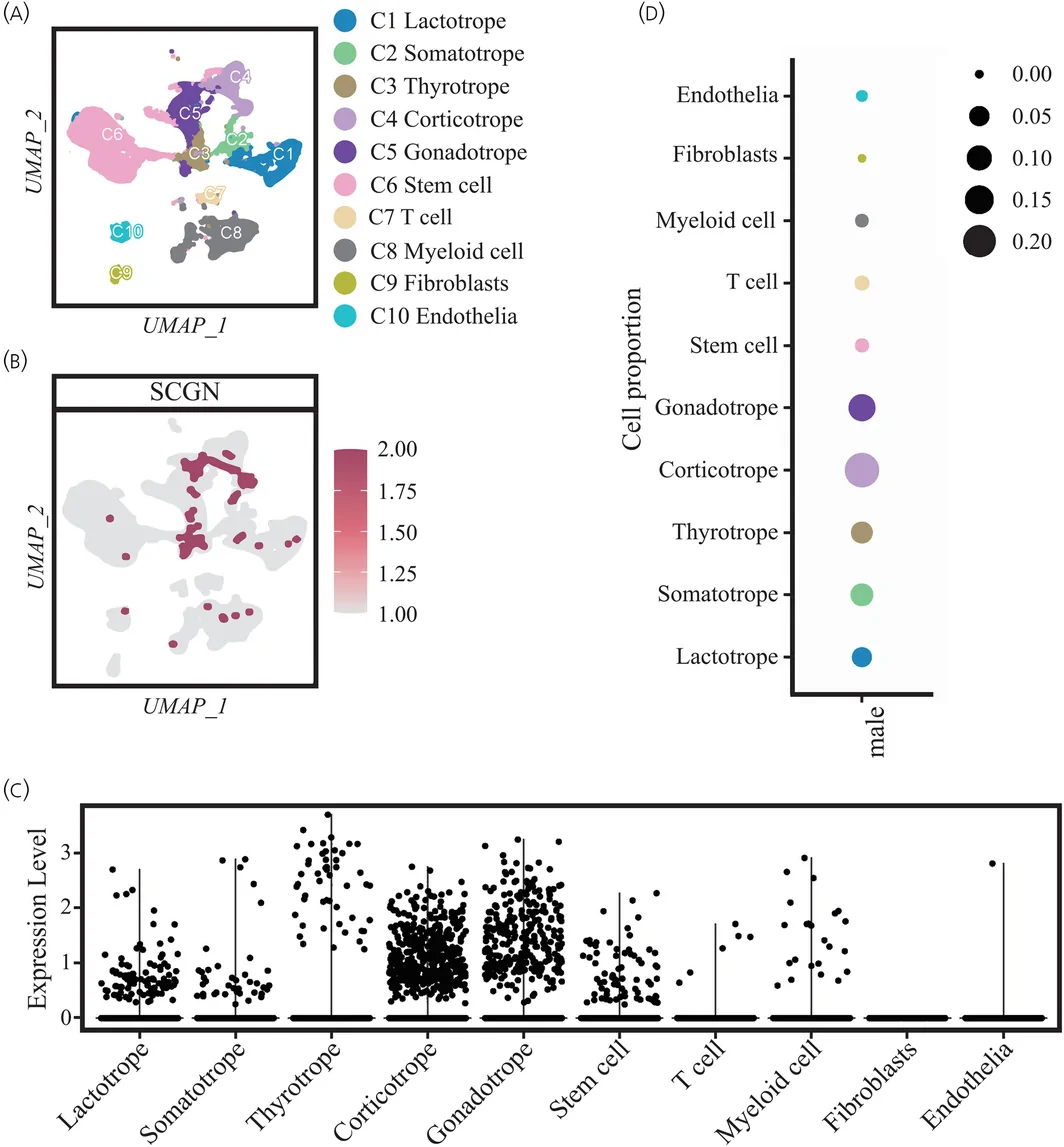
scRNA‐seq data analysis on the expression of SCGN in the anterior pituitary gland of male pig (*n* = 3). A: Anterior pituitary gland cell clusters identified by genetic markers and visualized with UMAP. B: SCGN in the anterior pituitary gland of pig at the cell level. C: Expression of SCGN in 10 types of male porcine anterior pituitary cells. D: SCGN positive cells of male pig. Circle represents the cell number percentage.

We performed scRNA‐seq on pituitary gland from 3‐month‐old normal male pigs. Cell populations were annotated and clustered based on the expression signatures of key transcription factors, identifying 10 distinct cell types in the anterior pituitary, including corticotropes, gonadotropes, lactotropes, somatotropes, and thyrotropes (Figure 4A). Enrichment analysis of SCGN transcript expression revealed that SCGN transcripts were primarily enriched in corticotropes and gonadotropes, with lower expression levels in other cell types (Figure 4B,C). Furthermore, quantification of the proportion of SCGN‐expressing cells across various anterior pituitary gland cell types in male pigs demonstrated that among SCGN‐positive cells, the proportions classified as corticotropes and gonadotropes were significantly higher than those of other cell types (Figure 4D), suggesting a specific expression trend of SCGN in TPIT‐lineage corticotropes and SF‐1‐lineage gonadotropes.

**FIGURE 5:**
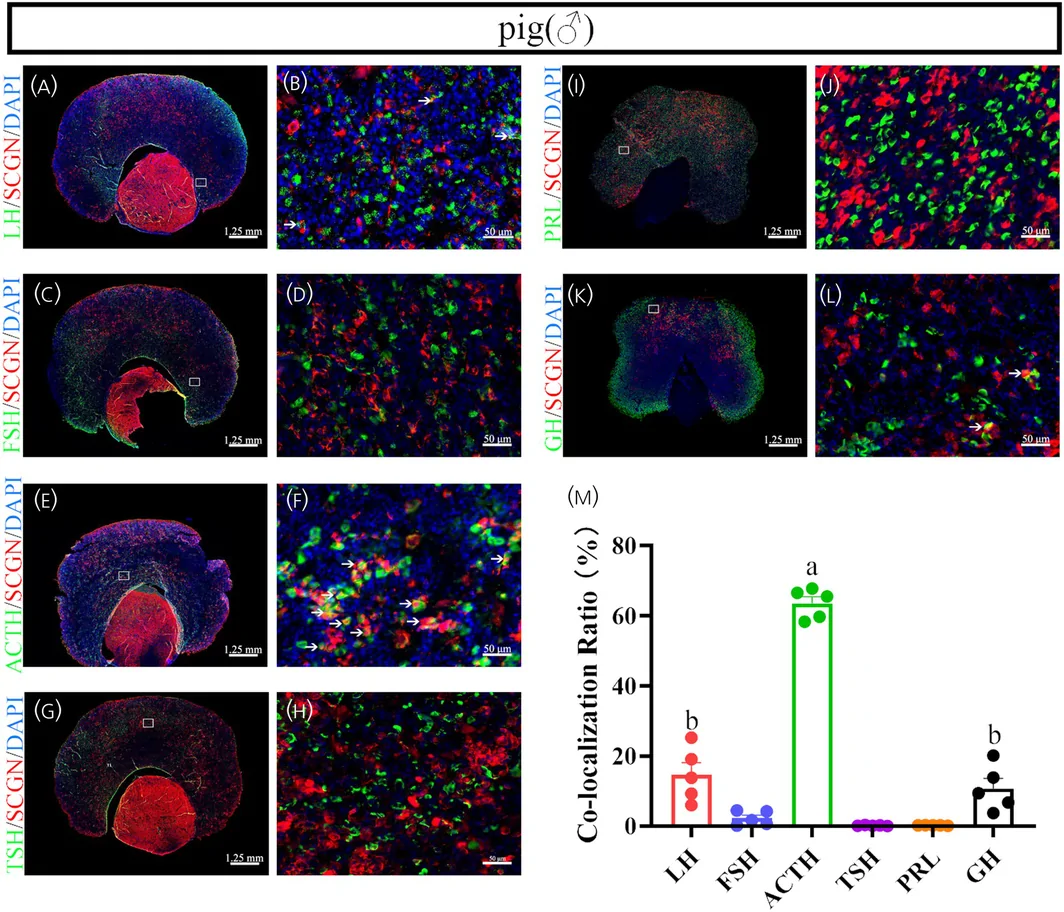
Immunofluorescence colocalization assays of SCGN in the anterior pituitary gland of 3‐month‐old male pig (*n* = 5). A,B: Colocalization of SCGN (red) and LH (green) in the anterior lobe (A), with a higher‐magnification view of the boxed area showing colocalization (arrows) (B). C,D: Localization of SCGN (red) and FSH (green) in anterior lobe (C), with a higher‐magnification view of the boxed area showing the detailed localization (D). E,F: Colocalization of SCGN (red) and ACTH (green) in anterior lobe (E), with a higher‐magnification view of the boxed area showing colocalization (arrows) (F). G,H: Localization of SCGN (red) and TSH (green) in anterior lobe (G), with a higher‐magnification view of the boxed area showing the detailed localization (H). I,J: Localization of SCGN (red) and PRL (green) in anterior lobe (I), with a higher‐magnification view of the boxed area showing the detailed localization (J). K,L: Colocalization of SCGN (red) and GH (green) in anterior lobe (K), with a higher‐magnification view of the boxed area showing colocalization (arrows) (L). Scale bars: 1.25 mm (A, C, E, G, I, K), 50 μm (B, D, F, H, J, L). Strong SCGN immunoreactivity observed in the posterior lobe. M: Quantitative analysis of colocalization rates between SCGN and individual anterior pituitary gland hormones in male pig. Data in the figure are mean ± SEM. Different letters above bars indicate significant differences (*p* < .05), bars sharing the same letter are not significantly different. One‐way ANOVA (arcsine square‐root transformed): F(5, 24) = 107.1, *p* < .0001. Welch’ s ANOVA (heteroscedasticity‐robust): W(5, 10.13) = 318.9, *p* < .0001.

To validate the expression distribution of SCGN at the protein level, we performed double immunofluorescence staining to analyze the colocalization of SCGN with six specific hormone markers in the anterior pituitary tissues of 3‐month‐old male pigs (Figure 5). Quantitative analysis of fluorescence colocalization revealed that SCGN had the highest colocalization rate with ACTH (63.581% ± 4.253%), followed by considerably lower rates with GH (10.759% ± 6.434%) and LH (14.688% ± 7.696%). In contrast, the colocalization with FSH (2.243% ± 1.992%), TSH (0.132% ± 0.118%), and PRL (0.200% ± 0.073%) was almost negligible (Figure 5M). These results indicate that SCGN is primarily and specifically localized in corticotropes. Notably, unlike the scRNA‐seq findings, the colocalization rate of SCGN with gonadotropes was relatively low. Collectively, integrating both single‐cell transcriptomic data and double immunofluorescence staining results, we confirmed that SCGN is primarily and specifically expressed in corticotropes in the anterior pituitary of male pigs.

### 3.3 SCGN expression is enriched in lactotropes, somatotropes, and thyrotropes in human anterior pituitary gland

**FIGURE 6:**
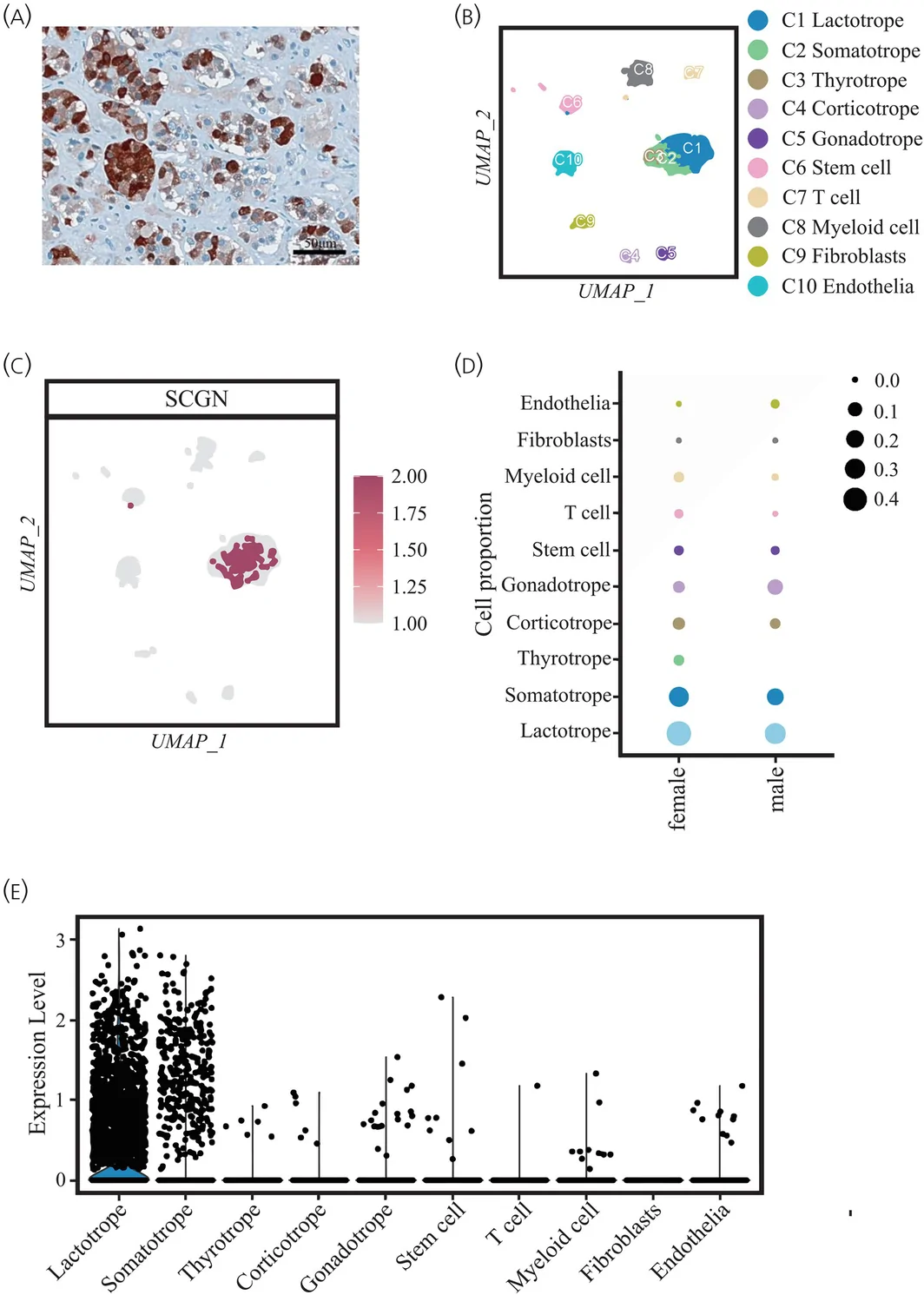
Immunohistochemistry and scRNA‐seq results of SCGN expression in the human anterior pituitary gland. A: Immunohistochemistry validation confirms SCGN expression in the human anterior pituitary gland (*n* = 3). B: Anterior pituitary gland cell clusters identified by genetic markers and visualized with UMAP. C: SCGN in the anterior pituitary gland of human at the cell level. D: SCGN positive cells of male and female (4 male, 3 female). Circle represents the cell number percentage. E: Expression of SCGN in 10 types of human anterior pituitary gland cells. Scale bars: 50 μm.

To investigate the expression pattern of SCGN in the human anterior pituitary gland, we first performed immunohistochemical staining on human anterior pituitary gland tissue sections. The IHC results confirmed SCGN expression at the histological level, with positive signals detected in the cytoplasm of anterior pituitary gland cells (Figure 6A).

Subsequently, we integrated and analyzed scRNA‐seq data from 4 male and 3 female human pituitary gland samples, which enabled annotation and clustering of cell populations based on key transcription factor expression signatures, identifying 10 major cell types including lactotropes, gonadotropes, somatotropes, thyrotropes, and corticotropes (Figure 6B). Subsequent enrichment analysis of SCGN transcript expression revealed significant enrichment in lactotropes and somatotropes, with minimal distribution observed in thyrotropes, gonadotropes, and corticotropes (Figure 6C–E). Further quantification of SCGN‐expressing cells across anterior pituitary cell types demonstrated similar proportional trends between sexes, with higher rates predominantly observed in lactotropes, somatotropes, thyrotropes, and gonadotropes (Figure 6D). Collectively, the scRNA‐seq data suggest that SCGN exhibits a specific expression trend within the human anterior pituitary, particularly in PIT1‐lineage lactotropes and somatotropes.

### 3.4 Absence of SCGN expression in the rat anterior pituitary gland

**FIGURE 7:**
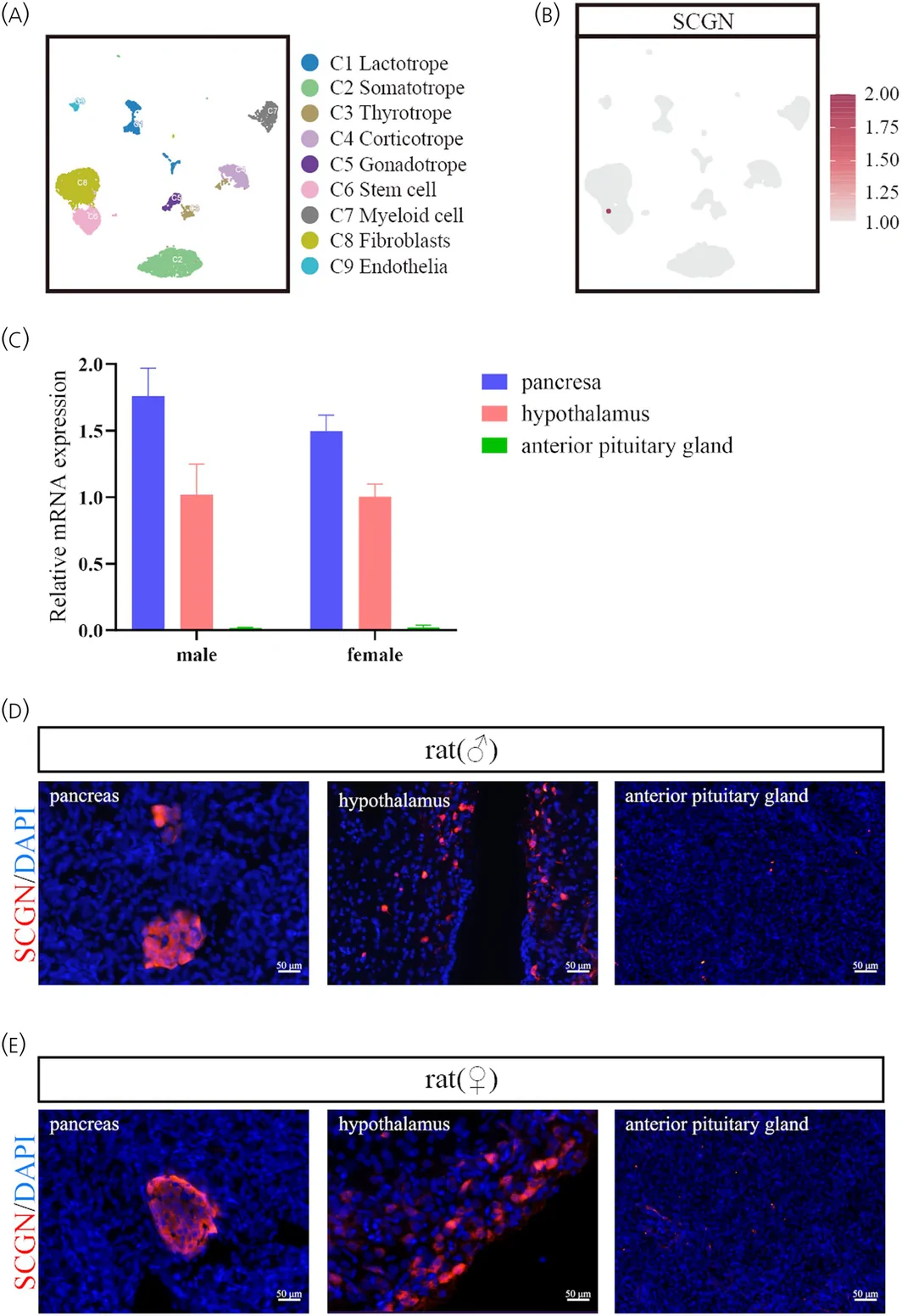
scRNA‐seq data and immunofluorescence analysis of SCGN expression in the anterior pituitary gland of rats. A: Anterior pituitary gland cell clusters identified by genetic markers and visualized with UMAP. B: SCGN in the anterior pituitary gland of rat at the cell level (*n* = 4). C: Relative SCGN mRNA expression in pancreas, hypothalamus and anterior pituitary gland of male and female rats measured by RT‐qPCR (*n* = 3). D: Immunofluorescent staining of SCGN (red) in male rat pancreas, hypothalamus and anterior pituitary gland. E: Immunofluorescent staining of SCGN (red) in female rat pancreas, hypothalamus and anterior pituitary gland. Scale bars: 50 μm.

By integrating publicly available scRNA‐seq data of the pituitary gland from SD rats, we annotated and clustered cell populations based on the expression signatures of key transcription factors, categorizing them into major types including lactotropes, gonadotropes, somatotropes, thyrotropes, and corticotropes (Figure 7A). Enrichment analysis of SCGN transcript expression revealed no significant enrichment of SCGN in any of these classified cell populations (Figure 7B). Meanwhile, we selected the pancreas and hypothalamus of rats as positive controls, as the presence of SCGN expression has been reported in these tissues.^24^, ^25^ The RT‐qPCR results showed that SCGN mRNA was detected at high transcription levels in the pancreas and hypothalamus of both male and female rats, but almost none was detected in the anterior pituitary gland (Figure 7C). Similarly, positive cytoplasmic immunostaining signals of SCGN were detected in the pancreas and hypothalamus of rats by immunofluorescent experiments, but no immunostaining signal was observed in the anterior pituitary region (Figure 7D,E).

## 4 DISCUSSION

Through integrated analysis of scRNA‐seq data from mice, rats, pigs, and human pituitaries, combined with immunofluorescence and immunohistochemical validation, this study reveals the expression and distribution patterns of SCGN in the anterior pituitary across multiple species. The results demonstrate remarkable species‐specific SCGN expression: it is predominantly enriched in PIT1‐lineage lactotropes and SF‐1‐lineage gonadotropes in mice; primarily localized to TPIT‐lineage corticotropes in pigs; and highly expressed in PIT1‐lineage lactotropes and somatotropes in humans. Notably, no SCGN expression was detected in the rat anterior pituitary gland. These findings reveal that SCGN expression is species‐restricted rather than universally conserved. Importantly, the complete absence of SCGN in the rat anterior pituitary suggests that SCGN is not essential for fundamental anterior pituitary hormone secretion, at least in this species.

The species‐specific differential expression of SCGN in the pituitary gland is not an isolated phenomenon. Multiple studies have demonstrated that the expression and distribution of certain regulatory peptides and hormone receptors also vary across species in the pituitary. For instance, galanin is primarily expressed in lactotropes of female rats, where it promotes prolactin secretion, whereas in male rats, it is mainly found in somatotropes, thyrotropes, and corticotropes.^26^ In adult male monkeys, galanin expression is more prominent in thyrotropes and gonadotropes, while in the normal human pituitary, it is almost exclusively restricted to corticotropes and exerts broader regulatory effects on multiple neuronal cell types.^27^, ^28^ Although mice and rats are both rodents, there are also other genes with similar species‐specific expression differences between the two. For example, the rat GnRHR promoter drives expression in the pituitary gland, hippocampus, and testes, while the mouse GnRHR is largely limited to the pituitary gland, and transgenic studies have shown that this difference is determined by the rat promoter itself.^29^ Thus, the absence of SCGN in rat and its cell‐type‐specific enrichment in mice, pigs, and humans might reflect species‐specific transcriptional regulation rather than coding sequence differences.

An increase in intracellular calcium concentration serves as the primary intracellular signal triggering the fusion of secretory vesicles with the plasma membrane for the release of hormones and neurotransmitters. Consequently, the presence of SCGN as a calcium‐sensing protein in different hormone‐secreting cell types suggests a potentially conserved mechanism: SCGN may sense intracellular calcium signals triggered by hypothalamic releasing hormones and interact with cytoskeletal and vesicle trafficking‐related proteins, thereby facilitating the directed transport and priming of secretory vesicles toward the plasma membrane.^10^, ^30^, ^31^, ^32^ The secretion of LH/FSH is mainly promoted by the binding of GnRH and its receptors in the hypothalamus, which facilitates the influx of extracellular Ca^2+^ and activates G protein, initiating downstream typical signaling pathways and regulating the biosynthesis and secretion of gonadotropins.^33^, ^34^, ^35^, ^36^ SCGN has been identified as a multifunctional calcium sensor that alternately binds SNAP‐25 and Syntaxin‐4 via its hydrophobic groove, acting as a temporal regulator during vesicle recruitment, docking, and release, while competitively inhibiting premature SNARE complex assembly. ^37^, ^38^, ^39^ Therefore, in gonadotropes, SCGN may bind SNAP‐25 under resting conditions to prevent excessive spontaneous vesicle fusion. Upon signal activation, Ca^2+^ influx may induce SCGN dissociation from SNAP‐25 or a conformational change, permitting efficient SNARE complex assembly and thus enabling precise, pulsatile release of LH and FSH. ACTH release is regulated by hypothalamic corticotropin‐releasing hormone (CRH) and occurs via Ca^2+^‐dependent exocytosis.^40^, ^41^ We thus speculate that SCGN may also participate in ACTH secretion by modulating the Ca^2+^‐SNAP‐25 axis. CRH induces Ca^2+^ influx, SCGN binds to Ca^2+^, undergoes conformational changes, and releases SNAP‐25, allowing for effective SNARE complex assembly and Ca^2+^‐triggered ACTH secretion. This mechanism is similar to the process of SCGN in insulin secretion at the molecular level, suggesting that SCGN could act as a Ca^2+^‐regulated exocytosis‐dependent promoter in cell types where it is expressed. Furthermore, studies have shown that SCGN is involved in CRH secretion regulation within neurons and participates in the systemic stress response pathway,^12^, ^42^, ^43^ suggesting that SCGN's role may extend beyond local pituitary regulation, potentially acting as a component coordinating the hypothalamic–pituitary–adrenal axis at multiple levels to modulate ACTH release. The expression of SCGN in PIT1‐lineage cells, particularly lactotropes, in both humans and mice, suggests its potential involvement in a fundamental secretion mechanism shared across this lineage. Beyond regulation by hypothalamic inhibiting and releasing factors, PRL synthesis and secretion are stimulated by various agents including dopamine, TRH, estradiol, and olfactory marker protein.^44^, ^45^, ^46^ Unlike gonadotropes, a defining secretory characteristic of lactotropes is their capacity for high basal hormone release. This is attributed to spontaneous plateau‐bursting action potentials that drive sustained, efficient voltage‐gated calcium influx, thereby continuously activating Ca^2+^‐dependent hormone exocytosis.^45^, ^47^ In this context, SCGN may maintain a readily releasable pool of secretory vesicles by stabilizing SNAP‐25. Additionally, by sensing the persistent calcium influx during action potential plateaus, SCGN could fine‐tune vesicle priming and release probability, supporting the high basal secretory activity of these cells. Meanwhile, the marked species‐specific distribution of SCGN suggests that it is not a universal or essential exocytotic factor. In species where it is expressed, SCGN could act as a cell‐type‐specific auxiliary modulator (or “gain control”) that fine‐tunes Ca^2+^‐triggered hormone release according to local physiological demands. SCGN enrichment in mouse gonadotropes may support rapid reproductive cycling; in pig corticotropes, it may underpin robust stress‐axis reactivity; in human lactotropes/somatotropes, it may align with sustained lactation and linear growth.

Regarding expression profile similarities, SCGN in the human pituitary gland is predominantly enriched in PIT1‐lineage‐derived lactotropes and somatotropes. Correspondingly, SCGN in the mouse pituitary gland is also distinctly enriched in PIT1‐lineage lactotropes. This key commonality suggests that mice and humans may share similar conserved SCGN‐dependent mechanisms in regulating hormone pathways related to growth, metabolism, and lactation. In contrast, in the porcine model, SCGN is specifically localized to TPIT‐lineage corticotropes. Given the highly overlapping PIT1‐lineage expression profiles between mice and humans, the mouse may serve as a useful model for studying SCGN expression and regulation. In species where SCGN is expressed, it could play a modulatory role in exocytosis. However, the absence of SCGN in the rat anterior pituitary argues against a critical, non‐redundant function, and any speculation about SCGN dysfunction leading to secretory deficiencies must be considered with caution. The specific expression of SCGN in pituitary cells across different species suggests that its expression may be regulated by specific transcription factors (e.g., PIT1, TPIT, SF‐1). Aberrant expression of these transcription factors themselves serves as a core marker for the molecular subtyping of pituitary adenomas. Therefore, SCGN may function as a downstream effector, mediating the tumor proliferation or secretory phenotypes driven by specific transcription factors.

In summary, SCGN exhibits species‐specific expression patterns in the anterior pituitary gland and is completely absent in the rat, suggesting SCGN may not be a universal core component in the exocytosis mechanism of the anterior pituitary. The expression of SCGN in the anterior pituitary gland seems to be evolutionarily different—existing in some mammalian lineages but unnecessary in others. Whether other calcium‐binding proteins compensate for the absence of SCGN in the rat pituitary, or whether SCGN is truly functionally redundant, remains an open question for future investigation. These findings challenge the assumption that SCGN plays a conserved role in endocrine function across mammals and underscore the importance of species‐specific considerations when extrapolating findings from animal models to human physiology.

## AUTHOR CONTRIBUTIONS

**Zhen‐Le Zang:** Investigation; funding acquisition; conceptualization. **Zhao‐Yun Zhang:** Writing – review and editing. **Cheng‐Cheng Wang:** Investigation; writing – original draft. **Yu Pan:** Investigation. **Hui Yang:** Writing – review and editing. **Yi‐Ming Li:** Resources. **Yi‐Fei Yu:** Resources. **Qi‐Lin Zhang:** Resources. **Hua‐Chun Yin:** Formal analysis; funding acquisition; data curation. **Song Li:** Writing – review and editing; conceptualization; funding acquisition.

## FUNDING INFORMATION

This work was supported by Natural Science Foundation of Chongqing (CSTB2024NSCQ‐MSX0021), Young Doctor Incubation Program of Xinqiao Hospital (2023YQB038, 2023YQB055, 2025YQB055), Chongqing Technology Innovation and Application Development Special Project (CSTB2024TIAD‐STX0044).

## CONFLICT OF INTEREST STATEMENT

The authors declare no conflicts of interest.

## ETHICS STATEMENT

This study has been approved by the Laboratory Animal Welfare and Ethics Committee of the Army Medical University (Approval No. AMUWEC20245310).

## Data Availability

The publicly available datasets analyzed during this research are accessible from the following sources: mouse pituitary gland data (GSM4594334, GSM4594335, GSM4594336, GSM4594338, GSM4594339) and rat pituitary gland data (GSE132224) from the NCBI GEO database; and human pituitary gland data (HRA002436, HRA003483) from the GSA database. The latter are subject to controlled access and can be requested through their respective Data Access Committees (DACs) as specified on the GSA repository pages. The scRNA‐seq data from the porcine pituitary gland generated in this study is available from the corresponding author upon reasonable request.

## References

[1] Alatzoglou KS , Gregory LC , Dattani MT . Development of the pituitary gland. Compr Physiol. 2020;10(2):389‐413. 32163208 10.1002/cphy.c150043

[2] Oh JY , Osorio RC , Jung J , et al. Transcriptomic profiles of Normal pituitary cells and pituitary neuroendocrine tumor cells. Cancers (Basel). 2022;15(1):110. 36612109 10.3390/cancers15010110 PMC9817686

[3] Zhang S , Cui Y , Ma X , et al. Single‐cell transcriptomics identifies divergent developmental lineage trajectories during human pituitary development. Nat Commun. 2020;11(1):5275. 33077725 10.1038/s41467-020-19012-4 PMC7572359

[4] Matsumoto R , Takahashi Y . Human pituitary development and application of iPSCs for pituitary disease. Cell Mol Life Sci. 2021;78(5):2069‐2079. 33206204 10.1007/s00018-020-03692-8 PMC11071979

[5] Beumer J , Puschhof J , Bauzá‐Martinez J , et al. High‐resolution mRNA and Secretome atlas of human Enteroendocrine cells. Cell. 2020;181(6):1291‐1306.e19. 32407674 10.1016/j.cell.2020.04.036

[6] Sharma AK , Khandelwal R , Sharma Y . Veiled potential of Secretagogin in diabetes: correlation or coincidence? Trends Endocrinol Metab. 2019;30(4):234‐243. 30772140 10.1016/j.tem.2019.01.007

[7] Wagner L , Oliyarnyk O , Gartner W , et al. Cloning and expression of secretagogin, a novel neuroendocrine‐ and pancreatic islet of Langerhans‐specific Ca^2+^‐binding protein. J Biol Chem. 2000;275(32):24740‐24751. 10811645 10.1074/jbc.M001974200

[8] Liu Z , Tan S , Zhou L , et al. SCGN deficiency is a risk factor for autism spectrum disorder. Signal Transduct Target Ther. 2023;8(1):3. 36588101 10.1038/s41392-022-01225-2 PMC9806109

[9] Lv Y , Xiao S , Ouyang S , et al. Ox‐LDL induces autophagy‐mediated apoptosis by suppressing secretagogin‐regulated autophagic flux in pancreatic beta‐cells. Acta Biochim Biophys Sin (Shanghai). 2022;54(12):1822‐1831. 36789686 10.3724/abbs.2022186 PMC10157621

[10] Maj M , Wagner L , Tretter V . 20 years of Secretagogin: exocytosis and beyond. Front Mol Neurosci. 2019;12:29. 30853888 10.3389/fnmol.2019.00029 PMC6396707

[11] Rogstam A , Linse S , Lindqvist A , James P , Wagner L , Berggård T . Binding of calcium ions and SNAP‐25 to the hexa EF‐hand protein secretagogin. Biochem J. 2007;401(1):353‐363. 16939418 10.1042/BJ20060918 PMC1698678

[12] Romanov RA , Alpár A , Zhang MD , et al. A secretagogin locus of the mammalian hypothalamus controls stress hormone release. EMBO J. 2015;34(1):36‐54. 25430741 10.15252/embj.201488977 PMC4291479

[13] Kosaka T , Kosaka K . Calcium‐binding protein, secretagogin, specifies the microcellular tegmental nucleus and intermediate and ventral parts of the cuneiform nucleus of the mouse and rat. Neurosci Res. 2018;134:30‐38. 29366872 10.1016/j.neures.2018.01.005

[14] Liu X , Liu X , Hu Y , et al. Secretagogin is highly expressed in Enteroendocrine K cells and plays a critical role in nutrient‐induced GIP secretion. J Endocr Soc. 2025;9(3):bvaf022. 40012909 10.1210/jendso/bvaf022 PMC11859952

[15] Tapia‐González S , Insausti R , DeFelipe J . Differential expression of secretagogin immunostaining in the hippocampal formation and the entorhinal and perirhinal cortices of humans, rats, and mice. J Comp Neurol. 2020;528(4):523‐541. 31512254 10.1002/cne.24773 PMC6972606

[16] Ruf‐Zamojski F , Zhang Z , Zamojski M , Smith G , Mendelev N , et al. Single nucleus multi‐omics regulatory atlas of the murine pituitary [scRNA‐Seq]; NCBI Gene Expression Omnibus. 2021; [dataset] GSE151958 (including samples GSM4594334, GSM4594335, GSM4594336, GSM4594338, GSM4594339).

[17] Fletcher PA , Iben J . [Dataset] Cell Type‐ and Sex‐Dependent Transcriptome Profiles of Rat Anterior Pituitary Cells; NCBI Gene Expression Omnibus; GSE132224. 2019. 10.3389/fendo.2019.00623 PMC6760010 31620083

[18] Yan N , Xie W , Wang D , et al. scRNA‐seq of Pituitary Tumor; Genome Sequence Archive; HRA003483 (Controlled access). 2023; [dataset].

[19] Zhang Q , Yao B , Long X , et al. Single‐Cell Characterization Identified the Origin and Novel Classification of Pituitary Neuroendocrine Tumors; Genome Sequence Archive; HRA002436 (Controlled access). 2022; [dataset].

[20] Fletcher PA , Smiljanic K , Maso Prévide R , et al. Cell type‐ and sex‐dependent transcriptome profiles of rat anterior pituitary cells. Front Endocrinol (Lausanne). 2019;10:623. doi:10.3389/fendo.2019.00623 31620083 PMC6760010

[21] Ruf‐Zamojski F , Zhang Z , Zamojski M , et al. Single nucleus multi‐omics regulatory landscape of the murine pituitary. Nat Commun. 2021;12(1):2677. 33976139 10.1038/s41467-021-22859-w PMC8113460

[22] Yan N , Xie W , Wang D , et al. Single‐cell transcriptomic analysis reveals tumor cell heterogeneity and immune microenvironment features of pituitary neuroendocrine tumors. Genome Med. 2024;16(1):2. doi:10.1186/s13073-023-01267-3 38167466 PMC10759356

[23] Zhang Q , Yao B , Long X , et al. Single‐cell sequencing identifies differentiation‐related markers for molecular classification and recurrence prediction of PitNET. Cell Rep Med. 2023;4(2):100934. doi:10.1016/j.xcrm.2023.100934 36754052 PMC9975294

[24] Gartner W , Vila G , Daneva T , et al. New functional aspects of the neuroendocrine marker secretagogin based on the characterization of its rat homolog. Am J Physiol Endocrinol Metab. 2007;293(1):E347‐E354. 17426113 10.1152/ajpendo.00055.2007

[25] Maj M , Milenkovic I , Bauer J , et al. Novel insights into the distribution and functional aspects of the calcium binding protein secretagogin from studies on rat brain and primary neuronal cell culture. Front Mol Neurosci. 2012;5:84. 22888312 10.3389/fnmol.2012.00084 PMC3412267

[26] Sato T , Yamaguchi T . Galanin in adrenocorticotropic hormone cells is decreased by castration. Cell Tissue Res. 2011;346(1):35‐41. 21975846 10.1007/s00441-011-1242-2

[27] Liu S . Galanin immunoreactive innervations of the anterior pituitary in the monkey and the dog. Brain Res. 2002;924(1):46‐55. 11743994 10.1016/s0006-8993(01)03010-4

[28] Tortorella C , Neri G , Nussdorfer GG . Galanin in the regulation of the hypothalamic‐pituitary‐adrenal axis (review). Int J Mol Med. 2007;19(4):639‐647. 17334639

[29] Ishaq M , Schang AL , Magre S , et al. Rat Gnrhr promoter directs species‐specific gene expression in the pituitary and testes of transgenic mice. J Mol Endocrinol. 2013;50(3):411‐426. 23536650 10.1530/JME-12-0231

[30] Yang SY , Lee JJ , Lee JH , et al. Secretagogin affects insulin secretion in pancreatic beta‐cells by regulating actin dynamics and focal adhesion. Biochem J. 2016;473(12):1791‐1803. 27095850 10.1042/BCJ20160137 PMC4901359

[31] Nussinovitch I . Ca2+ channels in anterior pituitary Somatotrophs: a therapeutic perspective. Endocrinology. 2018;159(12):4043‐4055. 30395240 10.1210/en.2018-00743

[32] Khandelwal R , Sharma AK , Chadalawada S , Sharma Y . Secretagogin is a redox‐responsive Ca2+ sensor. Biochemistry. 2017;56(2):411‐420. 27997125 10.1021/acs.biochem.6b00761

[33] Wang HQ , Ma YR , Zhang YX , et al. GnRH‐driven FTO‐mediated RNA m^6^A modification promotes gonadotropin synthesis and secretion. BMC Biol. 2024;22(1):104. 38702712 10.1186/s12915-024-01905-1 PMC11069278

[34] Stamatiades GA , Kaiser UB . Gonadotropin regulation by pulsatile GnRH: signaling and gene expression. Mol Cell Endocrinol. 2018;463:131‐141. 29102564 10.1016/j.mce.2017.10.015 PMC5812824

[35] Tzoupis H , Nteli A , Androutsou ME , Tselios T . Gonadotropin‐releasing hormone and GnRH receptor: structure, function and drug development. Curr Med Chem. 2020;27(36):6136‐6158. 31309882 10.2174/0929867326666190712165444

[36] Wang HQ , Zhang WD , Yuan B , Zhang JB . Advances in the regulation of mammalian follicle‐stimulating hormone secretion. Animals. 2021;11(4):1134. 33921032 10.3390/ani11041134 PMC8071398

[37] Lv Y , Xiang S , Cao R , Wu L , Yang J . SCGN‐regulated stage‐wise SNARE assembly: novel insight into synaptic exocytosis. Neurosci Bull. 2020;36(12):1576‐1578. 33025412 10.1007/s12264-020-00590-8 PMC7719123

[38] Qin J , Liu Q , Liu Z , et al. Structural and mechanistic insights into secretagogin‐mediated exocytosis. Proc Natl Acad Sci U S A. 2020;117(12):6559‐6570. 32156735 10.1073/pnas.1919698117 PMC7104245

[39] Szodorai E , Hevesi Z , Wagner L , Hökfelt TGM , Harkany T , Schnell R . A hydrophobic groove in secretagogin allows for alternate interactions with SNAP‐25 and syntaxin‐4 in endocrine tissues. Proc Natl Acad Sci U S A. 2024;121(16):e2309211121. 38593081 10.1073/pnas.2309211121 PMC11032447

[40] Kim DS , Chavera C , Gabor CR , Earley RL . Individual variation in ACTH‐induced cortisol levels in females of a livebearing fish at different gestational stages. Gen Comp Endocrinol. 2018;261:51‐58. 29374554 10.1016/j.ygcen.2018.01.022

[41] Stevens A , White A . ACTH: cellular peptide hormone synthesis and secretory pathways. Results Probl Cell Differ. 2010;50:63‐84. 19888563 10.1007/400_2009_30

[42] Romanov RA , Alpár A , Hökfelt T , Harkany T . Molecular diversity of corticotropin‐releasing hormone mRNA‐containing neurons in the hypothalamus. J Endocrinol. 2017;232(3):R161‐R172. 28057867 10.1530/JOE-16-0256

[43] Zhang M , Sun J , Wang Y , Tian Z . Secretagogin mediates the regulatory effect of Electroacupuncture on hypothalamic‐pituitary‐adrenal Axis dysfunction in surgical trauma. Neural Plast. 2021;2021:8881136. 33628224 10.1155/2021/8881136 PMC7880713

[44] Phillipps HR , Yip SH , Grattan DR . Patterns of prolactin secretion. Mol Cell Endocrinol. 2020;502:110679. 31843563 10.1016/j.mce.2019.110679

[45] Dobolyi A , Oláh S , Keller D , et al. Secretion and function of pituitary prolactin in evolutionary perspective. Front Neurosci. 2020;14:621. 32612510 10.3389/fnins.2020.00621 PMC7308720

[46] Kučka M , Gonzalez‐Iglesias AE , Tomić M , et al. Calcium‐prolactin secretion coupling in rat pituitary Lactotrophs is controlled by PI4‐kinase alpha. Front Endocrinol (Lausanne). 2021;12:790441. 35058881 10.3389/fendo.2021.790441 PMC8764672

[47] Van Goor F , Zivadinovic D , Martinez‐Fuentes AJ , Stojilkovic SS . Dependence of pituitary hormone secretion on the pattern of spontaneous voltage‐gated calcium influx. Cell type‐specific action potential secretion coupling. J Biol Chem. 2001;276(36):33840‐33846. 11457854 10.1074/jbc.M105386200

